# Distamycin A Inhibits HMGA1-Binding to the P-Selectin Promoter and Attenuates Lung and Liver Inflammation during Murine Endotoxemia

**DOI:** 10.1371/journal.pone.0010656

**Published:** 2010-05-14

**Authors:** Rebecca M. Baron, Silvia Lopez-Guzman, Dario F. Riascos, Alvaro A. Macias, Matthew D. Layne, Guiying Cheng, Cailin Harris, Su Wol Chung, Raymond Reeves, Ulrich H. von Andrian, Mark A. Perrella

**Affiliations:** 1 Division of Pulmonary and Critical Care Medicine, Brigham and Women's Hospital, Harvard Medical School, Boston, Massachusetts, United States of America; 2 Department of Medicine, Brigham and Women's Hospital, Harvard Medical School, Boston, Massachusetts, United States of America; 3 Department of Newborn Medicine, Brigham and Women's Hospital, Harvard Medical School, Boston, Massachusetts, United States of America; 4 Department of Physiological Sciences, Pontificia Universidad Javeriana, Bogota, Colombia; 5 Department of Biochemistry, Boston University School of Medicine, Boston, Massachusetts, United States of America; 6 CBR Institute for Biomedical Research and Department of Pathology, Harvard Medical School, Boston, Massachusetts, United States of America; 7 School of Biological Sciences, University of Ulsan, Ulsan, South Korea; 8 Department of Biochemistry and Biophysics, Washington State University, Pullman, Washington, United States of America; Emory University, United States of America

## Abstract

**Background:**

The architectural transcription factor High Mobility Group-A1 (HMGA1) binds to the minor groove of AT-rich DNA and forms transcription factor complexes (“enhanceosomes”) that upregulate expression of select genes within the inflammatory cascade during critical illness syndromes such as acute lung injury (ALI). AT-rich regions of DNA surround transcription factor binding sites in genes critical for the inflammatory response. Minor groove binding drugs (MGBs), such as Distamycin A (Dist A), interfere with AT-rich region DNA binding in a sequence and conformation-specific manner, and HMGA1 is one of the few transcription factors whose binding is inhibited by MGBs.

**Objectives:**

To determine whether MGBs exert beneficial effects during endotoxemia through attenuating tissue inflammation via interfering with HMGA1-DNA binding and modulating expression of adhesion molecules.

**Methodology/Principal Findings:**

Administration of Dist A significantly decreased lung and liver inflammation during murine endotoxemia. In intravital microscopy studies, Dist A attenuated neutrophil-endothelial interactions *in vivo* following an inflammatory stimulus. Endotoxin induction of P-selectin expression in lung and liver tissue and promoter activity in endothelial cells was significantly reduced by Dist A, while E-selectin induction was not significantly affected. Moreover, Dist A disrupted formation of an inducible complex containing NF-κB that binds an AT-rich region of the P-selectin promoter. Transfection studies demonstrated a critical role for HMGA1 in facilitating cytokine and NF-κB induction of P-selectin promoter activity, and Dist A inhibited binding of HMGA1 to this AT-rich region of the P-selectin promoter *in vivo*.

**Conclusions/Significance:**

We describe a novel targeted approach in modulating lung and liver inflammation *in vivo* during murine endotoxemia through decreasing binding of HMGA1 to a distinct AT-rich region of the P-selectin promoter. These studies highlight the ability of MGBs to function as molecular tools for dissecting transcriptional mechanisms *in vivo* and suggest alternative treatment approaches for critical illness.

## Introduction

Acute lung injury (ALI) represents a devastating clinical syndrome with increasing incidence that is initiated by an injurious stimulus, followed by the development of lung inflammation, increased alveolar-capillary barrier permeability, and influx of protein-rich edema fluid with resultant impairment in gas exchange due to alveolar flooding. Injury to the lung can be incurred through direct means (e.g., aspiration pneumonia), or, more commonly through indirect means (e.g., abdominal sepsis and resultant bacteremia often from gram negative rods that elaborate endotoxin). Despite the similar disruption of the alveolar-capillary membrane as an endpoint of both indirect and direct lung injury, the underlying mechanisms of injury are likely quite different, with direct injury initially targeting the lung alveolar epithelial cell and indirect injury activating the endothelium in the early stages [1]. Irrespective of the mechanism of lung injury, there exist no targeted treatment strategies for ALI, with current standard of care focusing on supportive approaches [2], [3]. Thus novel molecular strategies applied toward improving outcomes from ALI are desperately needed.

Transmigration of neutrophils into the lung represents a critical early pathophysiologic step in the development of ALI, as evidenced by ameliorated lung injury in some animal models in which neutrophils are eliminated [4], [5]. However, application of anti-inflammatory strategies (*e.g*., high-dose steroids, cyclooxygenase (COX-2) inhibitors) in ALI treatment has not proven universally effective [6], [7]. Possible reasons for failure of these approaches are multifactorial [8]–[10], including the inability to easily titrate a drug's effect, i.e., an “all or none” treatment effect. Therefore, targeted treatment approaches that modulate neutrophil migration in a titratable fashion during an inflammatory stimulus represent important avenues of investigation in ALI. One potential approach, which we examine herein, is to interfere predictably with transcription factor-DNA binding to target genes critical for neutrophil recruitment.

ALI frequently is triggered by bacterial infection, and there exist an important subset of patients who suffer infections by gram-negative bacteria. These pathogens elaborate endotoxin (lipopolysaccharide, LPS) that triggers a complex inflammatory cascade, including release of cytokines (*e.g*., tumor necrosis factor) and transcriptional up-regulation of numerous genes critical for the inflammatory response. A number of these cytokine-inducible genes share common regulatory elements in their promoter sequences and therefore are up-regulated by common transcription factors (TF). TFs such as NF-κB, IRF-1, and Stat-1 have been implicated as important regulators of a number of inducible genes in the inflammatory pathway, including P-selectin, E-selectin, vascular cell adhesion molecule (VCAM)-1, and nitric oxide synthase (NOS)-2 [11]–[16]. Specifically, P-selectin is upregulated by both LPS and TNF-α via similar transcriptional mechanisms [12], [17]–[19]. Beyond the simplified paradigm of TF-DNA binding leading to gene activation, elegant molecular studies have demonstrated that larger complexes of multiple transcription factors interacting with each other, as well as with the conserved DNA promoter motifs, are important for gene regulation and have been termed “enhanceosomes” [20].

Enhanceosome formation is facilitated by a group of proteins known as architectural transcription factors that are critical for gene regulation due to their ability to modify DNA conformation and to recruit DNA-binding of other TFs [21], [22]. High mobility group A1 (HMGA1, formerly known as HMG-I/Y) is an architectural transcription factor that binds to AT-rich DNA in the minor groove via three “AT-hook” DNA-binding motifs. HMGA1 binding sites are often adjacent to or overlap with consensus binding sites for conventional TFs. The role of HMGA1 in enhanceosome formation has been studied most extensively in regulating expression of the virus-inducible interferon (IFN)-β gene [20], [23], [24]. Similarly, HMGA1 plays a critical role in facilitating binding of nuclear factor (NF)-κB to the human E-selectin promoter [14], [15], and as demonstrated by our laboratory, to the NOS2 promoter [25], [26], to enable transcriptional up-regulation of these genes following inflammatory cytokine induction. Thus AT-rich regions surrounding TF consensus binding sites within promoters of a number of genes within the inflammatory cascade play critical roles in the inflammatory response. The ability to interfere with AT-rich region DNA binding in a predictable fashion therefore has significant potential to regulate a subset of genes and modulate the inflammatory response.

Minor groove binding drugs (MGBs), including the antibiotic Distamycin A (Dist A), constitute a class of drugs that bind AT-rich sequences within the minor groove of DNA in a sequence- and conformation-specific fashion, thereby interfering with TF-DNA binding to AT-rich sequences [27], [28]. HMGA1 is one of the few transcription factors known to bind exclusively to AT-rich DNA in the minor groove [29], [30]. Therefore, our group and other investigators have used MGBs to study the effect of interfering with HMGA1-DNA binding *in vitro* [26], [31], [32]. In order to determine whether MGBs might modulate gene expression in a similar fashion *in vivo*, we examined the effect of MGBs on mortality and hypotension during murine endotoxemia [33]. Dist A conferred a significant survival benefit following intraperitoneal LPS and attenuated the hypotensive response during murine endotoxemia. This beneficial effect *in vivo* correlated with attenuation of NOS2 induction in tissues and in murine macrophages. Furthermore MGBs interfered specifically with TF-DNA binding in a selective fashion to a distinct AT-rich region of the NOS2 enhancer. Thus, the ability to regulate transcription of targeted genes during an inflammatory state represents a novel and powerful tool toward development of potential therapeutics.

Given the presence of similar regulatory regions in the promoters of the inducible genes E-selectin and P-selectin and their roles in neutrophil recruitment to the tissues, we now hypothesize that MGBs might likewise affect transcriptional regulation of these genes. Thereby, attenuated neutrophil recruitment to the tissue might account for the beneficial effect of MGBs *in vivo*, and MGBs may therefore represent a new class of anti-inflammatory molecules. To test this hypothesis, we examined the effect of MGBs on neutrophil recruitment during murine endotoxemia. We analyzed the effect of MGBs on neutrophil-endothelial interactions *in vivo*, followed by testing of the effects of MGBs on expression and promoter trans-activation of candidate genes (P-selectin, E-selectin) involved in the distinct steps of the inflammatory cascade. Furthermore, the effects of MGBs on DNA-protein interactions were characterized and revealed that HMGA1-DNA binding is critical for full induction of the P-selectin promoter, and, moreover, that inhibition of HMGA1-DNA binding *in vivo* at a novel AT-rich DNA site within the P-selectin promoter correlates with attenuated inflammation during murine endotoxemia.

## Methods

### Murine endotoxemia

Male C57BL/6 wild-type (WT) mice (Charles River Laboratories, 6–8 weeks of age) were injected with lipopolysaccharide (LPS) 40 mg/kg (Escherichia coli serotype O26:B6 endotoxin, Sigma) or vehicle (saline) intraperitoneally (i.p.). Mice also received Distamycin A (25 mg/kg) i.p. 30 minutes prior to LPS administration (Dist A, Sigma) or Vehicle (dimethylsulfoxide, DMSO mixed with PBS, Sigma) as described previously [33]. RNA was extracted from lung tissue 2 hours following LPS treatment [33]. In separate experiments, lung and liver tissue was processed for immunohistochemistry between 4 and 24 hours after LPS treatment and stained for Gr-1 (neutrophils) (Pharmingen), or P-selectin (Santa Cruz Biotechnology) [34]. All research involving animals was conducted according to the recommendations for the “Guide for the Care and Use of Laboratory Animals”, and all animal studies were approved by the Harvard Medical Area Institutional Animal Care and Use Committee (IACUC). Animals were housed in pathogen-free barrier facilities and regularly monitored by the veterinary staff.

### Cell culture and reagents

Bovine aortic endothelial cells (BAEC) and primary murine lung endothelial cells (MLEC) (generous gift of Dr. Augustine Choi) were isolated and cultured as described previously [35], [36]. Murine bEnd.3 endothelial cells (American Type Culture Collection) were cultured as recommended. Human and murine recombinant tumor necrosis factor (TNF)-α were obtained from PeproTech Inc. (Rocky Hill, NJ).

### Plasmid constructs

The mouse [−1379/−13] P-selectin luciferase reporter plasmid (mp1379LUC, cloned into p0LUC) was a generous gift of Rodger P. McEver [11]. The human [−578/+35] E-selectin pCAT3 reporter plasmid was a generous gift of Tucker Collins [37]. The E-selectin promoter sequence was subcloned into the Acc65I/XhoI sites of pGL2-Basic (Promega) [34], resulting in generation of a plasmid construct termed (Esel-luc). The human dominant-negative HMGA1 cDNA construct (mutant HMGI(mII,mIII)) lacks the ability to bind AT-rich DNA sequences *in vitro* but retains capacity for specific protein-protein interactions with other transcription factors [38]. This mutant construct was subcloned into the HindIII/KpnI sites of the pCMVFlag expression vector (Sigma-Aldrich Co., St. Louis, MO) with optimization of the Kozak consensus sequence [39] ( CTTATG to GCC ATG), resulting in generation of a plasmid construct termed (DNHMGA1-pCMVFlag) [40]. The p50, p65, and HMGA1 expression vectors were generated through cloning full-length cDNA sequences [25] into pcDNA3 (Invitrogen). Constructs were confirmed by sequencing, and where appropriate, expression was tested using the TNT T7 Quick Coupled Transcription/Translation System (Promega Corporation, Madison, WI).

### Transient transfections of BAEC cells and reporter assays

P-selectin and E-selectin plasmids (1.0 µg) were transiently transfected into BAEC cells using FuGENE 6 transfection reagent (Roche Applied Science), as described previously [33]. Twelve hours following transfection of the reporter construct and a β-galactosidase expression vector (to normalize for luciferase activity), cells were conditioned in standard media containing 2% fetal bovine serum (FBS), then pre-treated with Dist A (25 µM) or Vehicle (ethanol, less than 1% final volume), followed by addition of LPS (1 µg/ml) or human TNF-α (10 ng/ml) 30 minutes later. Following treatment, cells were harvested 4 hours after LPS treatment and 12 hours after TNF-α treatment and assayed for luciferase activity (Promega Luciferase Assay System) and β-galactosidase [33]. In separate experiments, transient transfections were undertaken with the P-selectin promoter (0.5 µg), increasing concentrations of the DNHMGA1-pCMVFlag vector (0.5–1.0 µg, or an empty vector as a control), p50/p65 and HMGA1 expression vectors (0.25 µg each), and a β-galactosidase expression vector (to normalize for luciferase activity).

### RNA isolation and Northern blot analysis

RNeasy Mini RNA isolation kit (Qiagen) was used to extract total RNA from mouse tissues according to the manufacturer's instructions. Northern blot analysis using a radiolabeled murine P-selectin probe (generous gift of Rodger P. McEver [12]), E-selectin probe (generous gift of Mukesh Jain [41]), or HMGA1 probe [26], [42] was performed as previously described [33]. A radiolabeled rRNA 18S probe [33] was used to confirm equal loading. Quantitation of message for each gene relative to 18S was undertaken using ImageQuant software (GE Healthcare).

### Electrophoretic Mobility Shift Assay (EMSA)

EMSAs were performed as described previously [33] with double-stranded oligonucleotide probes encoding an AT-rich sequence within a region previously demonstrated to be critical for induction of the murine P-selectin promoter [(−542 to −521): 5′-AGAAATTCTCCCTGGATTTTCC-3′] [12]. Nuclear extracts were harvested from BAEC cells or primary murine lung endothelial cells with or without 1 hour of exposure to human TNF-α or murine TNF-α (10 ng/ml), and nuclear protein was quantified by the Bradford dye-binding method (Bio-Rad). HMGA1 peptide (43 amino acids) was synthesized by Tufts Physiology Dept Core Facility (Boston, MA) and encompassed the AT-hook DNA binding domains (DBD)-2 and DBD-3 [43]. The radiolabeled probes were incubated with 10–20 µM Dist A (or ethanol as a vehicle control) for two hours prior to electrophoresis. In separate experiments to test for presence of specific proteins within the TNF-α-inducible complex, the nuclear protein mixture was incubated for 30 min at room temperature with antibodies against NF-κB family members (p50 and p65, Santa Cruz), Ets-1 (unrelated antibody, Santa Cruz), or an isotype control IgG (Santa Cruz).

### Chromatin Immunoprecipitation (ChIP)

ChIP analysis was performed as described previously [44] using the Chromatin Immunoprecipitation Assay Kit (Millipore) on bEnd.3 cells. The protocol was carried out according to the manufacturer's instructions, using approximately 2×10^6^ cells harvested 3 hours after treatment with 10 ng/ml murine TNF-α and/or Dist A (50 µM, or the appropriate Vehicle control). Following formaldehyde crosslinking, cell lysates were sonicated 25 times for 15 sec each time to shear the genomic DNA to 200–1000 bp lengths. Immunoprecipitation was subsequently carried out with either an HMGA1 affinity-purified antibody [45] or an equivalent amount of rabbit IgG control. Following reversal of formaldehyde crosslinking, genomic DNA was purified using QIAquick PCR Purification Kit (Qiagen). For the positive control sample (“input”), a 1% volume of sample was removed before the immunoprecipitation step, followed by subsequent reversal of crosslinks and DNA purification as described. Precipitated (and “input” control) DNA was subjected to 35 cycles of PCR using primers to amplify a 246-basepair region of the murine P-selectin promoter (encompassing the AT-rich region at basepairs −542 to −521 described above) [basepairs −699 to −453; Forward primer: 5′-TCCTCTATCCATCTCTCTATCCATC-3′ ; Reverse primer: 5′-GGATGCCAGAGAATGGTTAAA-3′] or approximately 200-basepair regions of the murine P-selectin coding region and upstream promoter region as negative controls in vehicle-treated cells [Coding region-Forward primer: 5′-TGAAATCGCTCACCTCAATG-3′ ; Reverse primer: 5′-CCGCTTTCGTTTAAAACAGG-3′ ; Upstream promoter region-Forward primer: 5′-TTGTACCAACCTATGTAATTTCACAAC-3′; Reverse primer: 5′-GGGCCAAGCATTCTGTTAGT-3′]. Quantitation of precipitated DNA relative to input DNA was undertaken using Quantity One 1-D Analysis Software (Bio-Rad).

### Intravital Microscopy (IVM) and Image Analysis

IVM and analysis was performed as described previously [46]. Briefly, mice were treated with i.p. Dist A (25 mg/kg, or Vehicle control (DMSO/PBS)) followed 30 min later by intrascrotal murine TNF-α injection (500 ng). Mice were then anesthetized with i.p. ketamine/xylazine, and the right cremaster muscle was prepared as previously described [46] and overlaid with sterile, bicarbonate-buffered Ringer's injection solution (pH 7.4). Fluorescently labeled endogenous circulating leukocytes (achieved through injection of 2 mg/ml rhodamine 6G via internal jugular vein catheter insertion) were visualized by an X40 water-immersion objective (Zeiss Acroplan NA 0.75 ∞; Oberkochen, Germany) by video-triggered stroboscopic epi-illumination on an intravital microscope (IV-500; Mikron Instruments, San Marcos, CA). At least three venule trees per mouse were chosen, and 1-min recordings were collected of sub-segments of postcapillary and small collecting venules at 5-min intervals to assess baseline rolling. Measurements were taken hourly for four hours following TNF-α injection. The rolling fraction for each individual venule was calculated as percent leukocytes interacting with the vessel wall amongst total number of detected fluorescent cells passing the vessel during the observed period. Sticking efficiency is defined as the percentage of cells that engage in firm arrest (≥30 sec) among all leukocytes passing a microvessel during the analysis interval. Vessel cross-sectional diameters, velocities of individual rolling and non-interacting leukocytes, wall shear rate, and wall shear stress were determined as previously described [46].

### Statistical analysis

Results for each treatment group are summarized as mean values ± standard error (SE). Comparison of results among multiple groups at different time points was performed by two-way analysis of variance (StatMost Software, Salt Lake City, UT). Comparisons between means of two groups were performed using an unpaired t-test. Statistical significance was defined as a p value<0.05.

## Results

### Distamycin A Attenuates Lung and Liver Endotoxin-Induced Lung Inflammation

**Figure 1:**
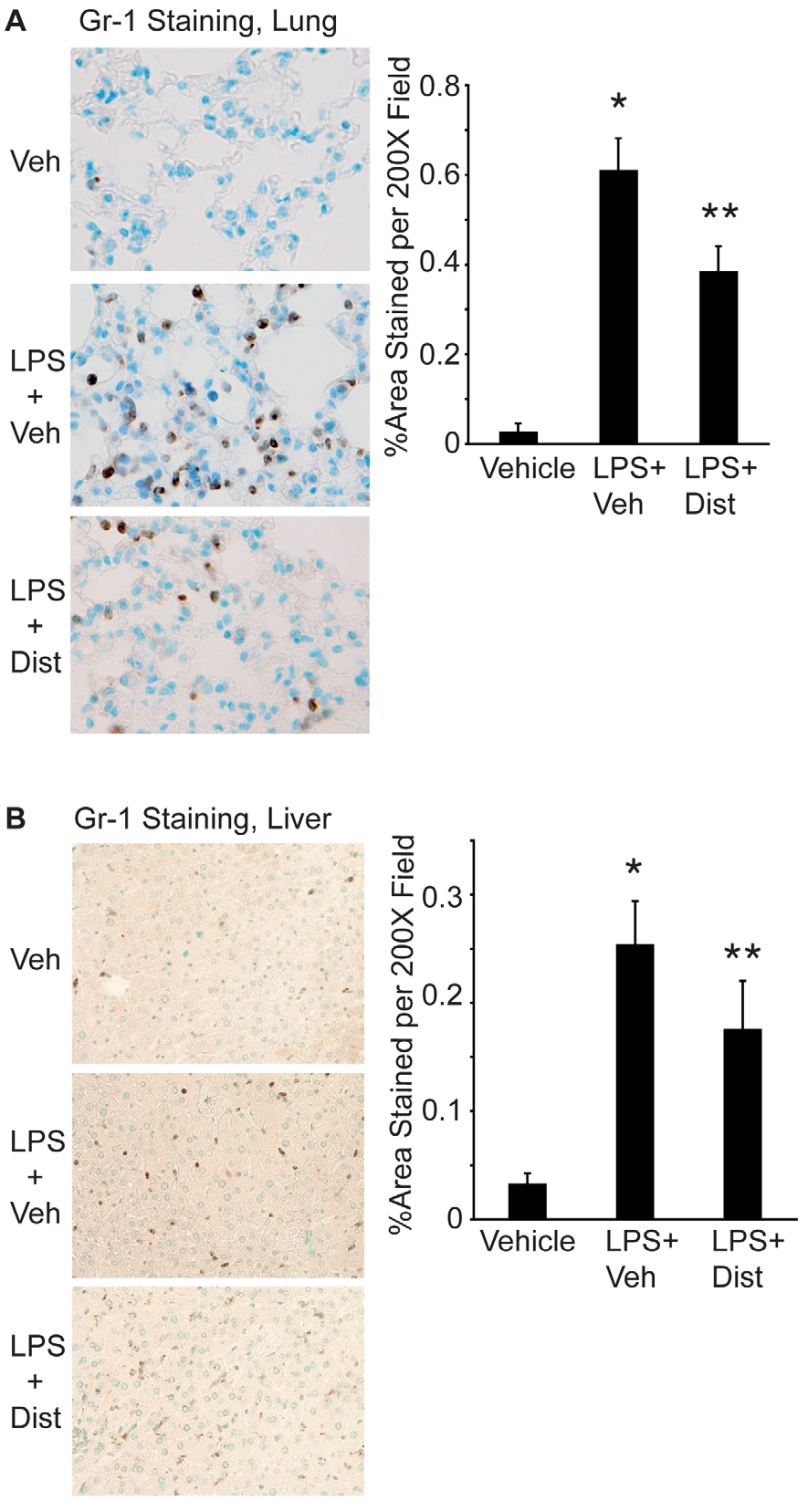
Distamycin A attenuates endotoxin-induced lung and liver inflammation. **A.** Lungs were harvested from C57BL/6 male mice 24 hours following treatment with Vehicle (Veh), LPS/Vehicle (LPS+Veh) or LPS/Dist A (LPS+Dist A) i.p. (n = 9 mice per group). Lungs were fixed, processed, sectioned, and stained for Gr-1 (a marker of neutrophils, positive staining indicated by brown cells). Representative sections for each condition are shown (200× magnification). Using NIH Image Software, 5 fields (at 200× magnification) per lung section from mice from each treatment group were quantified, with brown pixels counted as positive staining. Results were expressed as % positively stained area per 200× field. (*p<0.05 compared with Veh; **p<0.05 compared with LPS+Veh). **B.** Liver tissue was harvested from C57BL/6 mice 4 h after treatment with Vehicle (Veh), LPS/Vehicle (LPS+Veh) or LPS/Dist A (LPS+DistA) i.p., (n = at least 4 mice per group) then processed, stained for Gr-1 (neutrophil marker), and analyzed as described above for Fig. 1A. (*p<0.05 compared with Veh; **p<0.05 compared with LPS+Veh).

To examine the effect of Dist A on inflammation during systemic endotoxemia, C57BL/6 male adult mice were treated intraperitoneally (i.p.) with vehicle, LPS/Vehicle or LPS/Dist A (n = 9 per treatment group). Lung (Fig. 1A) and liver (Fig. 1B) tissue were harvested following treatment, processed for immunohistochemistry, and subjected to Gr-1 (neutrophil) staining. The number of positively stained cells in the Vehicle, LPS/Vehicle and LPS/Dist A groups was quantified using determination of brown pixilated area by NIH Image Software. As reported by our group and others, systemic endotoxin (LPS/Vehicle) results in recruitment of inflammatory cells to the lung interstitium [47], [48] and liver parenchyma [49]–[51] (p<0.05 compared with vehicle-treated mice in Fig. 1A–B). Interestingly, treatment with Dist A decreased endotoxin-induced lung inflammation at 4 hours following treatment, and this reduction remained at 24 hours following treatment (0.61±0.06 *vs* 0.38±0.05%Area per 200× field for LPS/Vehicle *vs* LPS/Dist A, respectively; p<0.05). Treatment with Dist A significantly decreased liver inflammation at four hours following treatment (0.25±0.04 *vs* 0.17±0.04%Area per 200× field for LPS/Vehicle *vs* LPS/Dist A, respectively, p<0.05), and this trend remained at 24 hours (0.16±0.1 *vs* 0.06±0.009%Area per 200× field for LPS/Vehicle *vs* LPS/Dist A, respectively), though with an overall reduction in Gr-1 staining in all groups at 24 hours compared with the 4-hour timepoint.

### Distamycin A Attenuates Inflammatory Cytokine-Induced Neutrophil-Endothelial Interactions

**Figure 2:**
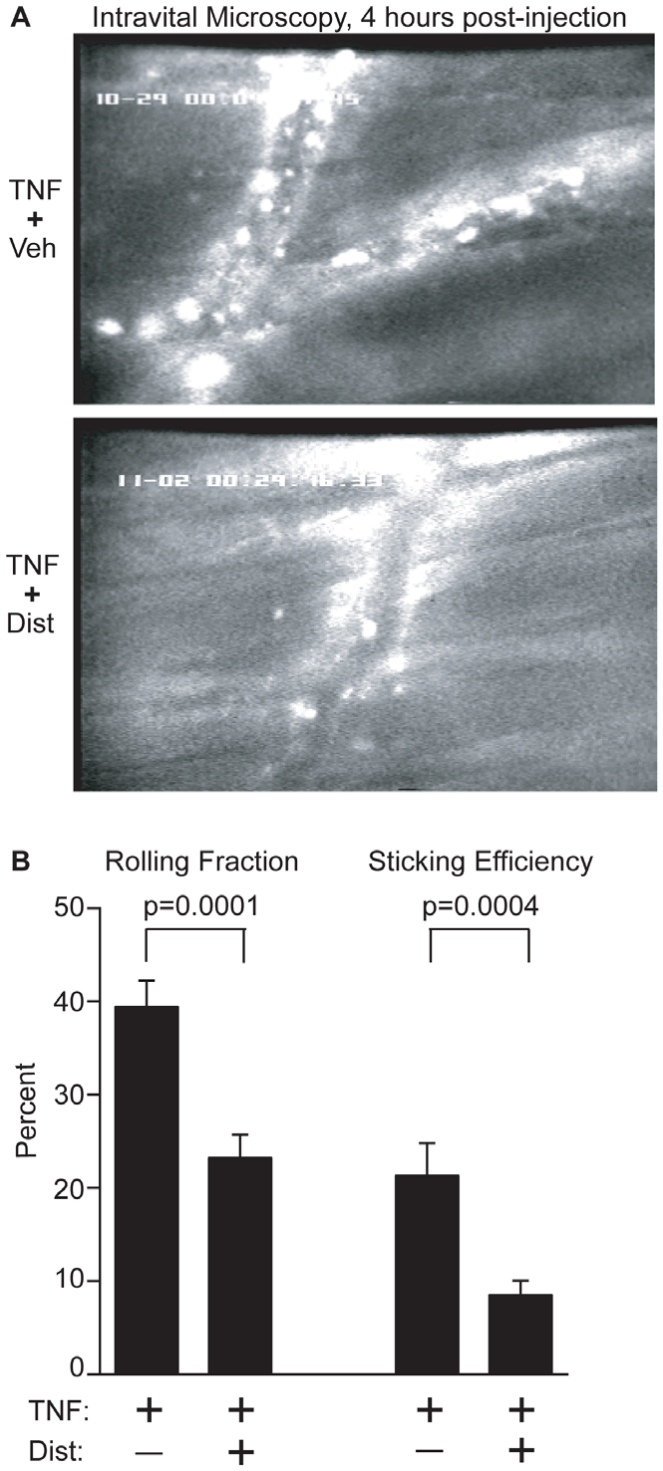
Distamycin A attenuates inflammatory cytokine-induced neutrophil-endothelial interactions. **A.** Wild type mice were subjected to intravital microscopy analysis at 3–4 hours following treatment with TNF-α/DistA or TNF-α/Vehicle (Veh) (n = 3 mice per treatment group with multiple vessels and vascular segments analyzed for each animal). Representative still photos of vascular segments are shown for the 4-hour time point. White round spots lining the vascular wall represent adherent leukocytes. **B.** Formal video analysis of the phases of neutrophil-endothelial interaction was performed, and values for all vascular segments within a particular treatment group were averaged for each time point. A significant reduction in rolling fraction and sticking efficiency was detected in the TNF-α/Dist A mice at 3–4 hours (p = 0.0001 and 0.0004, respectively), compared with the TNF-α/Vehicle mice.

We hypothesized that the effect of Dist A in attenuating endotoxin-induced lung and liver inflammatory cell recruitment would correlate with reduced interactions of circulating neutrophils with the endothelial surface. To test this hypothesis, we subjected mice to intravital microscopy of the cremasteric muscle at 3–4 hours following treatment with systemic TNF-α/Dist A or TNF-α/Vehicle (Representative Still Photos, n = 3 mice per treatment group, Figure 2A). TNF-α was selected for these experiments as representing a key pro-inflammatory cytokine in the LPS pathway. Preliminary experiments and intravital microscopy analysis demonstrated that total circulating cell counts were not altered by administration of Dist A alone (data not shown). However, the number of adherent cells to the endothelial surface was visibly reduced with the addition of DistA during a systemic inflammatory response, and formal analysis of the distinct phases of neutrophil-endothelial interaction [52] revealed a significant reduction in the rolling fraction (39.4±2.8% *vs* 23.2±2.5%, p = 0.0001) and sticking efficiency (21.3±3.5% *vs* 8.5±1.5%, p = 0.0004) in the TNF-α/Dist A-treated mice when compared with the TNF-α/Vehicle-treated mice (Fig. 2B).

### Distamycin A Selectively Decreases Induction of P-selectin Expression and Promoter Activity

**Figure 3:**
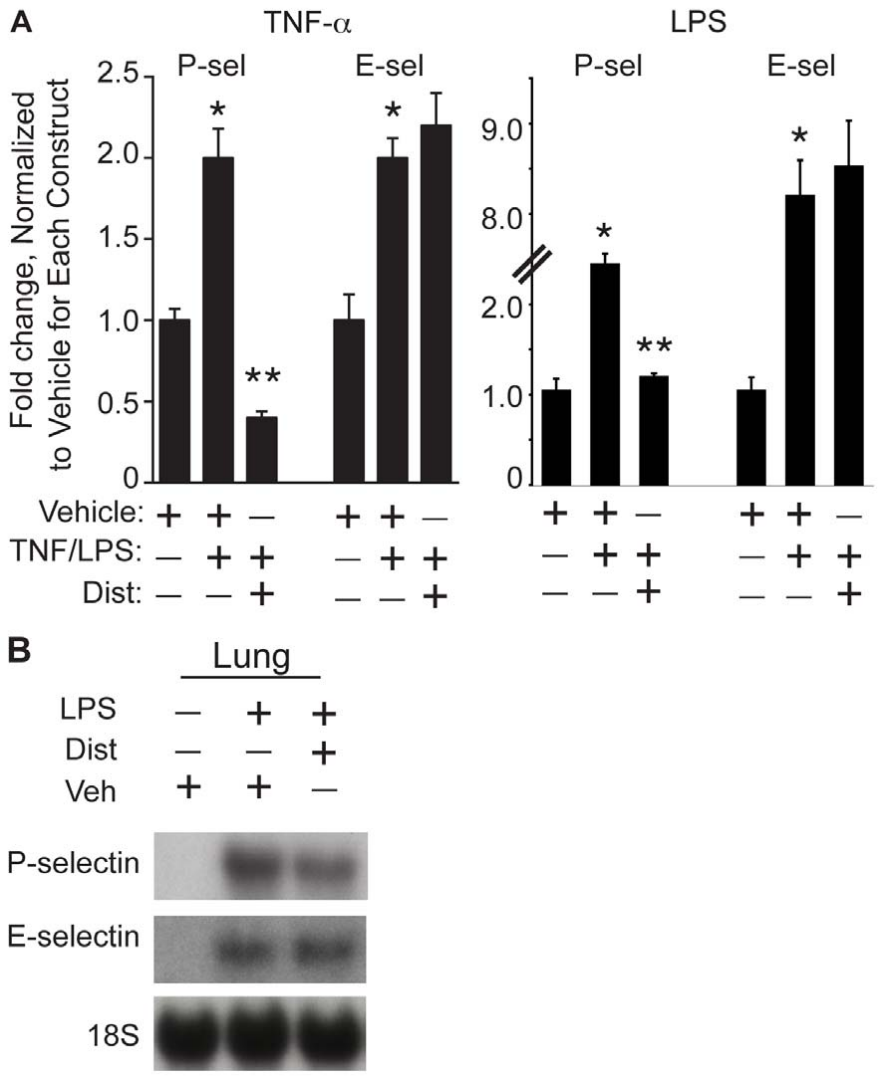
Distamycin A selectively decreases induction of P-selectin promoter activity and expression. **A.** BAEC cells were transiently transfected with promoter-reporter constructs for P-selectin and E-selectin. Transfected cells were treated with Vehicle, TNF-α/Vehicle, or TNF-α/Dist A, then harvested twelve hours after treatment and assessed for luciferase activity (with normalization for β-galactosidase levels). Similar experiments were performed in which transfected cells were treated with Vehicle, LPS/Vehicle, or LPS/Dist A and harvested four hours after treatment. Fold change was assessed relative to normalized values of “1” for the Vehicle-treated condition for each construct. These experiments were repeated 3 separate times with duplicate wells for each condition. (*p<0.05 compared with Vehicle for each construct; **p<0.05 compared with TNF-α/Vehicle or LPS/Vehicle for P-selectin). **B.** Lung tissue was harvested from wild type mice two hours following treatment with Vehicle (Veh), LPS/Vehicle, or LPS/Dist A (Dist), then subjected to RNA extraction and Northern blotting using a radiolabeled probe for P-selectin or E-selectin (and an 18S probe as loading control). This experiment was repeated two separate times.

Given the role of the minor groove binder Dist A in decreasing rolling fraction and sticking efficiency of leukocytes in TNF-α-treated mice, we hypothesized that inducible expression and promoter activity of cytokine-induced genes critical for this effect would be reduced in the presence of Dist A. In considering the molecules critical for early neutrophil-endothelial interactions [52], we selected two adhesion molecules demonstrated previously to be inducible by cytokines in endothelial cells: P-selectin [12] and E-selectin [53]. We hypothesized that reduction of P-selectin message would be mediated at the transcriptional level and, therefore, that induction of P-selectin promoter activity would be selectively attenuated by Dist A. To test the effect of Dist A on cytokine induction of P- and E-selectin promoter activities, we performed transient transfections in bovine aortic endothelial cells (BAEC) using promoter-reporter constructs for each of these genes. Transfected cells were then treated with Vehicle, TNF-α/Vehicle, or TNF-α/Dist A; or with Vehicle, LPS/Vehicle, or LPS/Dist A and assessed for luciferase activity (with normalization for β-galactosidase activity) twelve hours or four hours after transfection, respectively (Figure 3A). As anticipated [12], [53], both genes exhibited inducible promoter activity following treatment with TNF-α and LPS. Interestingly, while inducible promoter activity of E-selectin was not significantly altered with the addition of Dist A to TNF-α or LPS (p = NS), the induction of P-selectin promoter activity was markedly attenuated when transfected cells were treated with TNF-α/Dist A or LPS/Dist A as compared with TNF-α/Vehicle (78% reduction, p = 0.006) or LPS/Vehicle (52% reduction, p = 0.004). Given the critical importance of P-selectin in the rolling fraction and sticking efficiency phases of the adhesion cascade [52], [54], these findings raise the important possibility that Dist A mediates reduced neutrophil-endothelial interaction through transcriptional down-regulation of P-selectin promoter activity. Moreover, in contrast to P-selectin, and similar to the findings of other investigators [55], cytokine-induced E-selectin promoter activity is not significantly altered in the presence of Dist A. These results lend further support to the importance of P-selectin in mediating the observed effects of Dist A on attenuated neutrophil recruitment in our model.

To assess the effect of Dist A on inducible expression of these genes, lung tissues were harvested from mice at two hours following treatment with Vehicle, LPS/Vehicle, or LPS/Dist A. Tissues were then subjected to RNA extraction and Northern-blotting using radiolabeled P-selectin or E-selectin probes (and an 18S probe as a loading control) (Fig. 3B). While both P-selectin and E-selectin expression increased following LPS treatment (21.5±1.5 fold for P-selectin and 2.0±1.5-fold E-selectin), only P-selectin expression was substantially attenuated following Dist A treatment (26.0±3.2% reduction for P-selectin *vs* 4.0±0.005% reduction for E-selectin).

### Distamycin A Attenuates P-selectin Tissue Expression in Lung and Liver During Endotoxemia

**Figure 4:**
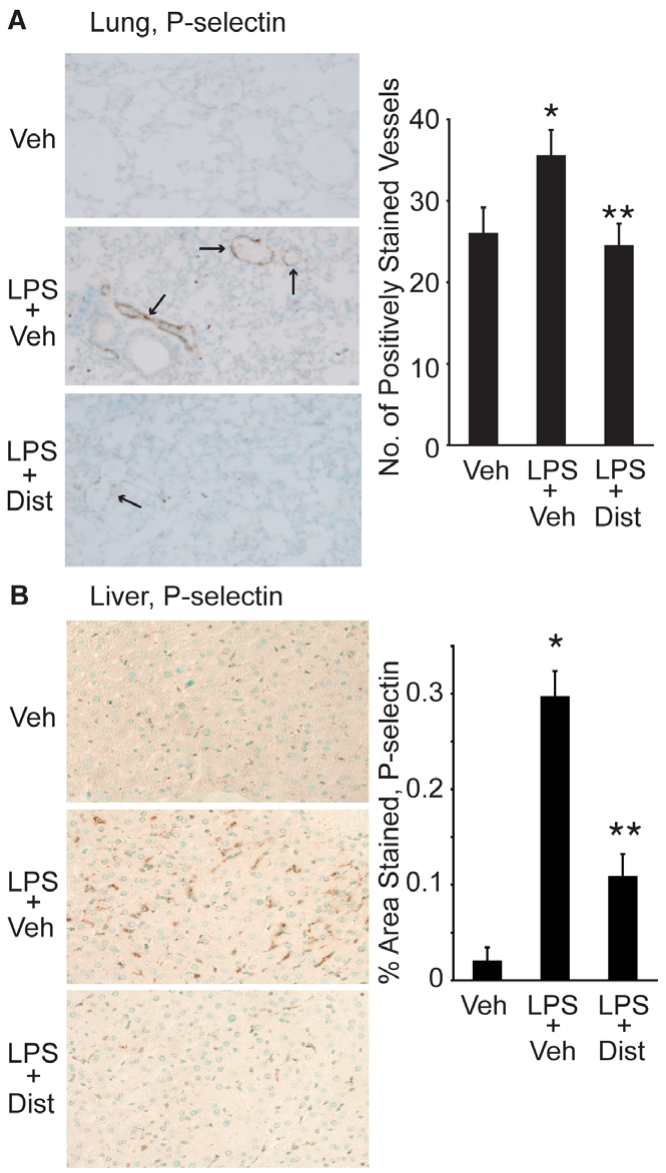
Distamycin A decreases lung and liver P-selectin tissue staining. **A.** Representative sections (200× magnification) of lung tissue harvested from C57BL/6 mice 24 h after treatment with Vehicle (Veh), LPS+Veh, or LPS+Dist A (Dist). Tissue was fixed, processed, and stained with a P-selectin antibody. Number of positively stained vessels was counted in sections from at least 5 mice in each treatment group. (Arrows demonstrate examples of positively stained vessels; *p<0.05 compared with Veh; **p<0.05 compared with LPS/Veh). **B.** Representative sections (200× magnification) of liver tissue harvested from mice 4 h after treatment with Vehicle (Veh), LPS+Vehicle (Veh) or LPS+Dist A (Dist). Tissue was fixed, processed, and stained with a P-selectin antibody. Using NIH Image Software, 5 fields (at 200× magnification) per liver section from mice from each treatment group were quantified, with brown pixels counted as positive staining. Results were expressed as % positively stained area per 200× field. (*p<0.05 compared with Veh; **p<0.05 compared with LPS/Veh).

Given the presence of P-selectin expression within vascular endothelial cells as well as within platelets, we examined lung and liver tissue sections for the effect of Dist A on inducible P-selectin expression within the lung and liver parenchyma that has been described by others during endotoxemia [49]–[51]. Lung and liver tissue was harvested from mice following treatment with Vehicle, LPS/Vehicle, or LPS/DistA. Lung (Fig. 4A) and liver (Fig. 4B) tissues were then subjected to immunohistochemistry using a P-selectin antibody. Analysis of lung sections revealed a significant LPS-induced increase in P-selectin staining within the lung vasculature that was reduced in the presence of Dist A at 4 hours after treatment, and this reduction remained at 24 hours after treatment (p<0.05 compared with LPS/Vehicle slides). Similarly, significant reduction in P-selectin staining in the liver was seen with LPS/DistA compared with LPS alone at 4 hours after treatment (0.29±0.02 *vs* 0.11±0.02%Area per 200× field for LPS/Vehicle *vs* LPS/Dist A, respectively, p<0.05). By 24 hours after treatment, this trend persisted (0.07±0.04 *vs* 0.01±0.005%Area per 200× field for LPS/Vehicle *vs* LPS/Dist A, respectively, p = NS). Notably, there was a significant overall reduction of P-selectin staining in all groups at 24 hours after treatment, compared with the four-hour timepoint.

### Distamycin A disrupts binding of an inducible protein-DNA complex containing NF-κB to an AT-rich region of the P-selectin promoter

**Figure 5:**
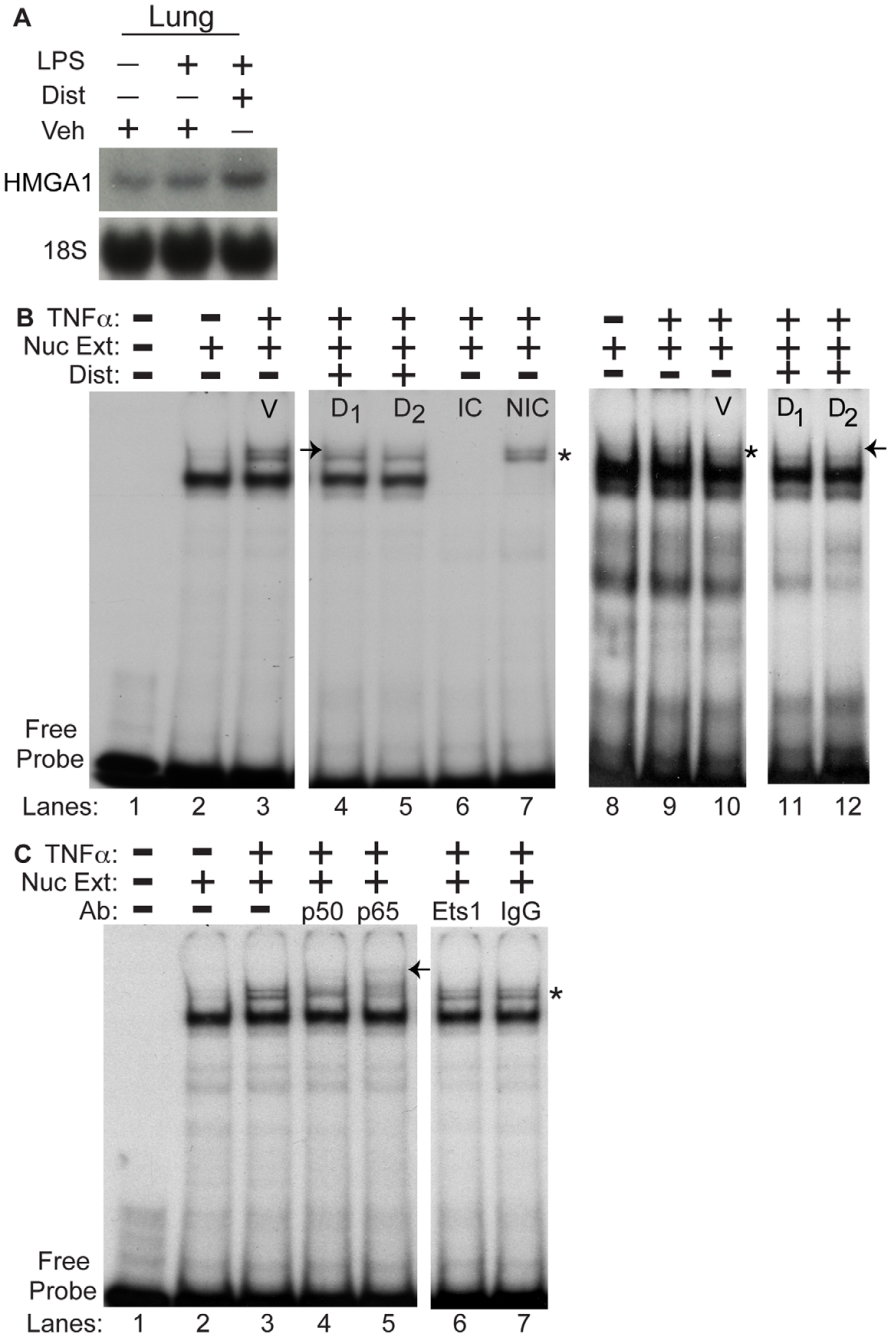
Distamycin A disrupts binding of an inducible protein-DNA complex containing NF-κB to an AT-rich region of the P-selectin promoter. **A.** As in Fig. 3B, lung tissue was harvested from wild type mice two hours following treatment with Vehicle (Veh), LPS/Vehicle, or LPS/Dist A (Dist), then subjected to RNA extraction. The same blot from Fig. 3B was hybridized with a radiolabeled probe for HMGA1 (and an 18S probe as loading control). This experiment was repeated two separate times. **B.** Nuclear extracts from BAEC cells (Lanes 1–7) and primary murine lung endothelial cells (MLEC, Lanes 8–12) (“Nuc Ext”) with (Lanes 3–7, 9–12) or without (Lanes 2,8) TNF-α stimulation were subjected to electrophoretic mobility shift assays (EMSA) using a radiolabeled probe spanning the AT-rich region of the P-selectin promoter (basepairs −542 to −521). Lane 1 represents the radiolabeled probe without addition of nuclear extract. TNF-α-treated nuclear extract was additionally incubated and electrophoresed with the radiolabeled probe and vehicle (V, lanes 3 and 10 (or in the presence of TNF-α without vehicle, Lane 9)) or increasing concentrations of Dist A (D_1_ = 10 µM, D_2_ = 20 µM, Lanes 4–5, 11–12) as well as with an identical competitor (IC, Lane 6) and a non-identical competitor (NIC, Lane 7). (* represents the inducible, specific complex seen following TNF-α treatment; “→” represents disruption of the TNF-α-inducible complex following addition of Dist A (Lanes 4–5 compared with Lane 3 and Lanes 11–12 compared with Lane 9–10). All of the binding studies were repeated at least two separate times. **C.** Nuclear extracts from BAEC cells (“Nuc Ext”) with (Lanes 3–7) or without (Lane 2) TNF-α stimulation were subjected to electrophoretic mobility shift assays (EMSA) using a radiolabeled probe spanning the AT-rich region of the P-selectin promoter (basepairs −542 to −521). Lane 1 represents the radiolabeled probe without addition of nuclear extract. TNF-α-treated nuclear extract was additionally incubated and electrophoresed with the radiolabeled probe and antibodies to the NF-κB family members p50 and p65 (lanes 4–5), or unrelated and control antibodies (Ets-1 and IgG control respectively, lanes 6–7). (* represents the inducible, specific complex seen following TNF-α treatment; “←” represents supershifted band/disruption of the TNF-α-inducible complex following addition of p50 and p65 antibodies (Lanes 4-5 compared with Lanes 6–7). All of the binding studies were repeated at least two separate times.

Given the effects of the minor groove binder Dist A in inhibiting inducible P-selectin expression in tissues and promoter-activity in endothelial cells, we next tested the hypothesis that Dist A decreases protein-DNA binding to the P-selectin promoter. We first examined HMGA1 expression in lung tissue of mice following vehicle, LPS/vehicle, or LPS/DistA to determine whether Dist A directly affected HMGA1 message levels (Fig. 5A). We hybridized the same blot as in Fig. 3B for HMGA1, and we found no significant effect of LPS or DistA on HMGA1 expression (1.25±0.075 fold change for LPS/veh *vs* vehicle and 1.31±0.25 fold change for LPS/DistA *vs* vehicle, respectively; p = NS). Next, to test DNA-protein binding activity, we examined an AT-rich DNA region within the P-selectin promoter that has previously been demonstrated to be critical for induction of promoter activity (basepairs −542 to −521). Pan *et. al.* demonstrated that members of the NF-κB family (p50/p65 subunits) are part of an inducible binding complex that forms within this promoter region [12]. Moreover, these authors speculated that HMGA1 might also bind this AT-rich region and facilitate NF-κB-binding through a mechanism similar to that described for upregulation of a number of other genes in the inflammatory cascade, including NOS2 and E-selectin [13], [14], [20], [24], [25]. We therefore set out to determine whether Dist A would disrupt a previously described inducible binding complex that forms at an AT-rich region of the P-selectin promoter and, moreover, whether HMGA1 binds with NF-κB family members to this AT-rich region.

Nuclear extracts harvested from BAEC cells or primary murine lung endothelial cells (MLEC) two hours following treatment with vehicle or TNF-α (human TNF-α for BAECs and murine TNF-α for MLECs) were electrophoresed with radiolabeled probes spanning the AT-rich region (basepairs −542 to −521) from the P-selectin promoter (Figure 5). As described previously [12], an inducible “doublet” complex was observed in BAEC and MLEC cells following treatment with TNF-α (Fig. 5B lanes 3 and 9–10 and Fig. 5C, lane 3). Next, Dist A (or Vehicle control, V) was incubated with the radiolabeled probe prior to gel electrophoresis. Dist A decreased binding within the TNF-α-inducible complex, particularly the upper band of the “doublet” (Fig. 5B, lanes 4-5, 11–12), and use of identical and non-identical cold competitors (IC, NIC) confirmed the specificity of this inducible complex, with elimination of the binding complex with the IC (Fig. 5B, lane 6) and retention of the inducible complex with the NIC (Fig. 5B, lane 7).

Incubation of the nuclear extracts from TNF-α-treated cells with NF-κB family member antibodies (p50 and p65) revealed presence of these proteins in the complex as indicated by supershifted and/or disrupted bands (Fig. 5C, lanes 4–5), while no supershift or disruption of the binding complex was observed with an unrelated antibody (Ets-1, Fig. 5C, lane 6) or an isotype control antibody (IgG, Fig. 5C, lane 7).

### HMGA1 is critical for induction of P-selectin promoter activity

**Figure 6:**
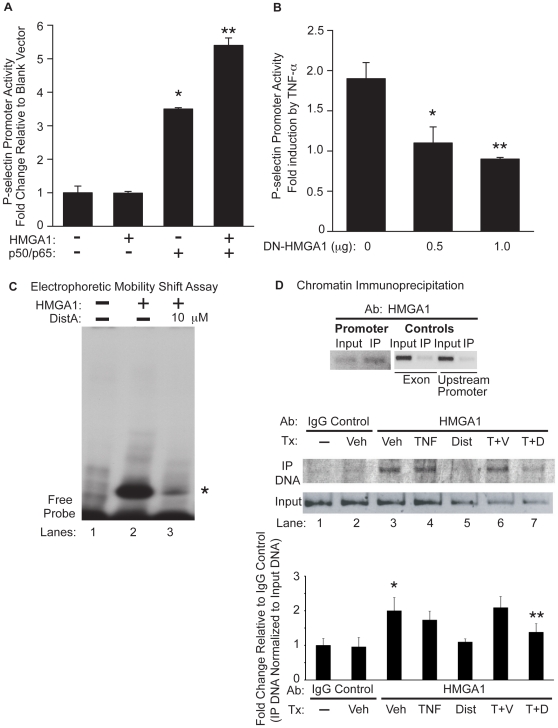
HMGA1 binds to the P-selectin promoter and is critical for full induction of P-selectin promoter activity. **A.** BAEC cells were transiently transfected with a P-selectin promoter-reporter construct with the addition of a blank expression vector, an expression vector for HMGA1, and/or expression vectors for NF-κB family members (p50/p65). Transfected cells were harvested and assessed for luciferase activity (normalized for β-galactosidase content). Results are expressed as fold-change in luciferase activity relative to transfection with the P-selectin promoter and a blank expression vector. **B.** BAEC cells were transiently transfected with a P-selectin promoter-reporter construct and increasing concentrations of a vector expressing a dominant-negative form of HMGA1 (DN-HMGA1). Transfected cells were stimulated with TNF-α, then harvested and assessed for luciferase activity (normalized for β-galactosidase content). Results are expressed for each transfection condition as fold-change in luciferase activity as a result of TNF-α stimulation. (*p<0.05 for 0.5 µg of DN-HMGA1 as compared with empty vector control; **p<0.05 for 1.0 µg of DN-HMGA1 as compared with empty vector control). This experiment was repeated three separate times, with each condition performed in triplicate. **C.** An electrophoretic mobility shift assay (EMSA) was performed using the HMGA1 peptide and a radiolabeled probe spanning the AT-rich region of the P-selectin promoter (basepairs −542 to −521) without (Lane 2) or with Dist A (10 µM, Lane 3). Lane 1 represents the radiolabeled probe in the absence of incubation with protein. (* represents the HMGA1-DNA complex in Lane 2 which is diminished in intensity following addition of Dist A in Lane 3). Binding studies were repeated at least two separate times. **D**. Chromatin Immunoprecipitation (ChIP) was performed on murine bEnd.3 endothelial cells without (Lane 1) or with 3 hours of exposure to Vehicle (Veh, Upper Panel and Lanes 2–3 Lower Panel), mTNF-α (TNF, Lane 4), Distamycin A (Dist A, Lane 5), mTNF-α/Vehicle (T+V, Lane 6), and mTNF-α/Dist A (T+D, Lane 7). Immunoprecipitation of crosslinked, sonicated cell lysates was carried out with an affinity-purified HMGA1 antibody (Upper panel and Lanes 3–7 Lower Panel) or IgG rabbit control antibody (Lanes 1–2 Lower Panel), with PCR amplification using primers spanning a 246-basepair AT-rich region of the P-selectin promoter (including basepairs −542 to −521; “Promoter” in Upper Panel and all lanes in Lower Panel) or 200-basepair fragments spanning a coding region or upstream promoter region of P-selectin (“Exon” and “Upstream Promoter”, respectively in Upper Panel; see sequences in Methods section). PCR amplification was undertaken for immunoprecipitated DNA as well as for “input” DNA as an additional control for each condition. Quantitation of precipitated DNA relative to input DNA was undertaken using Quantity One 1-D Analysis Software (Bio-Rad). This experiment was performed two separate times. (*p<0.05, for increase in binding for Veh (HMGA1 Ab) compared with IgG control; **p<0.05, for reduction in binding for TNF-α/Dist compared with TNF-α/Vehicle).

Given that Dist A selectively decreases P-selectin expression and promoter activity (Fig. 3–4) and, moreover, that Dist A disrupts formation of an inducible binding complex at an AT-rich region of the P-selectin promoter containing HMGA1 and NF-κB (Fig. 5), we hypothesized that HMGA1 binds to the P-selectin promoter in this region and plays a critical role in P-selectin induction. Conversely, inhibition of HMGA1 binding would therefore be expected to attenuate induction of P-selectin promoter activity. To test this hypothesis, we examined whether HMGA1 would facilitate NF-κB induction of the P-selectin promoter (Fig. 6A) and, conversely, whether a dominant-negative form of HMGA1 that does not bind DNA (DN-HMGA1) [38] would decrease TNF-α-induced P-selectin promoter activity (Fig. 6B).

BAEC cells were first transiently transfected with a P-selectin promoter-reporter construct with addition of expression vectors for NF-κB family members (p50/p65) and HMGA1 (Fig. 6A). No significant change in basal P-selectin promoter activity was seen with addition of HMGA1 alone, as has been described previously with other genes [25]. Addition of p50/p65 resulted in significant upregulation of the P-selectin promoter (p<0.05 *vs*. P-selectin promoter alone). Interestingly, the addition of HMGA1 along with p50/p65 resulted in synergistic upregulation of the P-selectin promoter, beyond that of p50/p65 alone (p<0.05 *vs*. P-selectin promoter alone). Thus, HMGA1 facilitates upregulation of P-selectin promoter activity by p50/p65.

To investigate the role of HMGA1 in upregulating P-selectin promoter activity, BAEC cells were transiently transfected with a P-selectin promoter-reporter construct and increasing concentrations of DN-HMGA1 (or empty vector control) (Fig. 6B). Transfected cells were treated with TNF-α, then harvested to assess for P-selectin promoter activity. We observed a significant reduction in TNF-α-induced P-selectin promoter activity in a dose-dependent fashion with increasing concentrations of DN-HMGA1 (p<0.05 for 0.5 µg and 1.0 µg DN-HMGA1 when compared with empty vector control). Of note, the DN-HMGA1 construct had no significant effect on the promoter activity of another unrelated gene (heme oxygenase-1, data not shown). Thus, HMGA1 is critical for TNF-α-induced P-selectin promoter activity.

### Distamycin A blocks *in vitro* and *in vivo* HMGA1 binding to a distinct AT-rich region of the P-selectin promoter

We next set out to further test the hypothesis that Dist A attenuates induction of P-selectin promoter activity through inhibiting binding of HMGA1 to an AT-rich DNA region. To do this, we carried out electrophoretic mobility shift assays (EMSAs) using a synthesized protein (to assess *in vitro* binding, Fig. 6C) and chromatin immunoprecipitation experiments using murine endothelial cells (to assess *in vivo* binding, Fig. 6D). First, EMSAs were performed using HMGA1 peptide (containing the AT-hook DNA binding domains, see Methods) and a radiolabeled probe spanning the AT-rich region of the P-selectin promoter (basepairs −542 to −521, as above). Significant binding of the HMGA1 peptide to this site was observed (Fig. 6C, lane 2), while addition of Dist A significantly reduced *in vitro* binding of the HMGA1 peptide (Fig. 6C, lane 3).

Next, to examine *in vivo* binding of HMGA1 to the AT-rich region of the P-selectin promoter, chromatin immunoprecipitation (ChIP) was performed using murine endothelial cells (bEnd.3) and an affinity-purified HMGA1 antibody [45]. We and others have described that HMGA1 can function as an architectural transcription factor that binds constitutively to AT-rich DNA. With cytokine treatment, HMGA1 then facilitates binding of other transcription factors to form an inducible transcription factor complex, or enhanceosome [16]. Therefore, we hypothesized that HMGA1 would bind the P-selectin promoter pre- and post-TNF-α treatment, while addition of Dist A would inhibit HMGA1 binding *in vivo*. To test this hypothesis, ChIP was performed on cells treated with vehicle, TNF-α/Vehicle, and TNF-α/Dist A (Fig. 6D). Immunoprecipitated DNA (as well as “input” control DNA) was amplified by PCR with primers spanning the AT-region of the P-selectin promoter described above, with presence of a band indicating *in vivo* binding of the designated protein to the amplified DNA segment. No significant *in vivo* DNA-protein binding was detected with use of the IgG control antibody (Fig. 6D, lanes 1–2 in lower panel) or in immunoprecipitated (IP) DNA with primers amplifying an upstream promoter region or coding region of the P-selectin gene in vehicle-treated cells (upper panel, Fig. 6D). In contrast, HMGA1 *in vivo* binding was detected in cells treated with Vehicle (“Promoter” in upper panel and lane 3 in lower panel; p<0.05 compared with IgG control Ab), TNF-α (lane 4, lower panel), and TNF-α/Vehicle (lane 6, lower panel). Interestingly, addition of Dist A (in the presence or absence of TNF-α, lanes 5,7 in lower panel) inhibited *in vivo* HMGA1-DNA binding to the designated AT-rich region of the P-selectin promoter (35.0±4.9% reduction in binding for TNF-α/Dist compared with TNF-α/Vehicle, p<0.05).

## Discussion

This study reports three important new findings. First, we present data supporting a novel anti-inflammatory strategy *in vivo* through using a minor groove binding drug to specifically interfere with DNA-protein binding in a targeted manner. Second, we demonstrate an important role for the architectural transcription factor HMGA1 in facilitating full induction of P-selectin promoter transactivation and inflammatory-induced gene expression. Third, we demonstrate that using transcriptional regulation to target select, similarly regulated inducible genes with common promoter motifs can be effective in improving outcomes in murine models of critical illness. Furthermore, our data supports the intriguing concept that minor groove binders can serve as an important *in vivo* tool to dissect molecular mechanisms of inflammatory disease processes.

Our previous work employed MGBs *in vitro* [25], [26], [33], [42] and *in vivo* [33] to confirm an important role for TF-binding to AT-rich DNA regions in transactivation of the NOS2 promoter and in attenuating mortality and hypotension during murine endotoxemia. Numerous genes critical for the inflammatory cascade share similar promoter regulatory regions with NOS2, and we therefore hypothesized that MGBs would attenuate the inflammatory response through similarly interfering with TF-binding to AT-rich DNA regions of promoters of genes critical for the inflammatory response. Recruitment of inflammatory cells to the tissue occurs via a complicated cascade of events, each step of which is highly regulated [52]. Leukocytes traversing the vasculature at rapid speed are lured to the activated endothelium through initial tethering and rolling mediated predominantly by members of the selectin family of adhesion molecules (P-selectin, E-selectin, and L-selectin). Leukocytes are then activated with subsequent firm adhesion to the endothelial surface facilitated in large part through interaction of immunoglobulin family members (on endothelial cell surface) with integrins (on leukocytes). Transmigration of leukocytes across the endothelial surface and into the tissue is likely mediated by a gradient of chemoattractants.

Given the role of MGBs in attenuating inducible gene expression [33] and the observed reduction of rolling fraction and sticking efficiency of leukocytes to the endothelial surface (Figure 2), we focused our studies on inducibly expressed genes previously demonstrated to play a role in the early tethering and rolling phases of leukocyte-endothelial surfaces. Therefore, we examined the effects of MGBs on P-selectin and E-selectin, both of which are inducibly expressed in endothelial cells ([52], Figure 3). Of note, L-selectin is constitutively expressed on leukocytes [56]. While numerous members of the adhesion molecule families provide a contributory role toward leukocyte sticking and rolling [54], [56]–[61], studies of knockout animals have revealed that P-selectin plays the most critical and pronounced role in early leukocyte tethering and rolling [54], [62]. Therefore, the fact that induction of P-selectin can be selectively attenuated in this model (Figure 3) represents an important example of the potential *in vivo* benefits of exquisite transcriptional control. Similar to the observed effects of MGBs on NOS2 in this model [33], inflammatory cell recruitment and P-selectin induction was decreased but not entirely abolished with MGB treatment (Figure 3). Interestingly, other investigators have reported that NOS2 does not play a role in regulating expression of endothelial adhesion molecules, suggesting that our findings regarding P-selectin are independent of the regulation of NOS2 expression by Dist A in this model [63]. Furthermore, P-selectin exhibits constitutive as well as inducible expression within endothelial cells [11], [12] such that the effect of leukocyte-endothelial interactions attributable to P-selectin is reduced but not eliminated with MGBs. Thus, the ability to selectively control gene transcription through interfering with TF-DNA binding presents the potential for a “titratable” anti-inflammatory effect, versus the “all or none” effect of more traditional anti-inflammatory approaches.

Interestingly, P-selectin is fairly unique as an adhesion molecule, as it is expressed not only in endothelial cells, but also in platelets, and roles of P-selectin in different locations has been a matter of recent debate. While numerous studies reported an important role for endothelial P-selectin expression in lung and liver inflammation and physiologic injury during endotoxemia [49]–[51], more recently, interest has arisen in the importance of platelet P-selectin expression in development of acid-induced lung injury [64]. While we cannot fully exclude the role of platelet P-selectin in our studies, examination of histologic sections (Figure 4) and our studies in endothelial cells *in vitro* (Figures 5–6) support a significant contribution of endothelial P-selectin to our observations. Moreover, a recent study reported that P-selectin glycoprotein-ligand-1 regulates lung neutrophil recruitment independently of circulating platelets in a murine model of abdominal sepsis (cecal ligation and puncture model [65]). In aggregate, our findings in conjunction with those in the literature support the intriguing possibility that mechanisms of tissue neutrophil recruitment during indirect lung injury (e.g., systemic endotoxin, abdominal sepsis) are distinct from those that predominate during direct injury. Tissue neutrophil recruitment during indirect lung injury might rely more heavily on endothelial P-selectin expression, while platelet P-selectin expression may play a more prominent role during direct lung injury.

Our results indicate that MGBs can serve as a useful tool to probe the functional effects of targeted DNA sequences in complicated biological systems *in vivo*. Through observing the effects of MGBs in inhibiting HMGA1-binding to a targeted AT-rich region of the P-selectin promoter and in demonstrating the effects of the dominant-negative HMGA1 construct in attenuating induction of the P-selectin promoter by TNF-α, we were able to derive an important role for HMGA1 in regulating P-selectin promoter induction (Figures 5–6). We acknowledge that examination of the effect of MGBs on P-selectin induction in the presence of HMGA1 knockdown would provide more direct evidence that HMGA1 expression is essential for the observed effects of DistA. However, these experiments were not technically feasible, given the interference of the siRNA reagents with the MGB agents.

Other investigators have elegantly demonstrated in Drosophila that targeted minor-groove binding drugs can be fed to flies that interfere with binding of the Drosophila HMGA1 orthologue (termed D1) to specific AT-rich DNA sequences and result in specific gain- and loss-of-function phenotypes [66]–[69]. In these studies, small cell-permeable molecules were synthesized based upon the existing known structure of Distamycin A and were designed with the goal of developing improved tools to elucidate the role of architectural DNA regions in biology in model systems [66]. In recent years, there has been increasing interest in development of derivatives of MGBs as human chemotherapeutics to allow targeted delivery of DNA-modifying agents [69], [70]. Furthermore, improvements in techniques to elucidate molecular structure has led to a growing literature on detailed characterization of MGB-DNA binding as well as on development of novel compounds with optimized DNA-binding and functional properties [71]–[75]. Our data supports the premise that novel small molecules interfering in a targeted way with sequence- and conformation-specific DNA binding can be studied at the molecular and physiologic level in higher order organisms subjected to models of human disease. Such approaches hold promise for development of novel treatment strategies for critical illness. To our knowledge, the present study and our prior work [33], [43] represent the first *in vivo* applications of MGBs to murine models of critical illness.

In summary, we now demonstrate that MGBs can interfere in a targeted manner with HMGA1 binding to the P-selectin promoter *in vivo*, resulting in attenuated P-selectin induction and decreased lung and liver inflammation during murine endotoxemia. These findings, in combination with our previous data showing improvement in mortality and hypotension during murine endotoxemia attributed to attenuated NOS2 induction [33], supports the interesting possibility that there exist select genes regulated by common promoter motifs that can be advantageously regulated to improve outcomes from critical illness. With a growing appreciation of conserved regulatory motifs throughout the human genome and with increasing ability to catalogue this data [76], there exists a real possibility of molecularly targeted treatment strategies that can be applied in individualized ways to complex human disease. We acknowledge that MGBs may have other effects on an organism that remain to be characterized. However, our work represents implementation of MGBs as a molecular tool to derive *in vivo* biological characterization of critical illness that ultimately can be applied to the development of novel therapeutics in a field where effective treatment approaches are desperately needed.

## References

[1] Matute-Bello G, Frevert CW, Martin TR (2008). Animal models of acute lung injury.. Am J Physiol Lung Cell Mol Physiol.

[2] The Acute Respiratory Distress Syndrome Network (2000). Ventilation with lower tidal volumes as compared with traditional tidal volumes for acute lung injury and the acute respiratory distress syndrome.. N Engl J Med.

[3] The National Heart, Lung, and Blood Institute Acute Respiratory Distress Syndrome (ARDS) Clinical Trials Network (2006). Comparison of two fluid-management strategies in acute lung injury.. N Engl J Med.

[4] Abraham E (2003). Neutrophils and acute lung injury.. Crit Care Med.

[5] Abraham E, Carmody A, Shenkar R, Arcaroli J (2000). Neutrophils as early immunologic effectors in hemorrhage- or endotoxemia-induced acute lung injury.. Am J Physiol Lung Cell Mol Physiol.

[6] Ware LB, Matthay MA (2000). The acute respiratory distress syndrome.. N Engl J Med.

[7] MacCallum TS, Evans TW (2005). Epidemiology of acute lung injury.. Curr Opin Crit Care.

[8] Lewis JF, Brackenbury A (2003). Role of exogenous surfactant in acute lung injury.. Crit Care Med.

[9] Bone RC (1996). Sir Isaac Newton, sepsis, SIRS, and CARS.. Crit Care Med.

[10] Marshall JC (1999). Rethinking sepsis: from concepts to syndromes to diseases.. Sepsis.

[11] Pan J, Xia L, McEver RP (1998). Comparison of promoters for the murine and human P-selectin genes suggests species-specific and conserved mechanisms for transcriptional regulation in endothelial cells.. J Biol Chem.

[12] Pan J, Xia L, Yao L, McEver RP (1998). Tumor necrosis factor-α or lipopolysaccharide-induced expression of the murine P-selectin gene in endothelial cells involves novel κB sites and a variant activating transcription factor/cAMP response element.. J Biol Chem.

[13] Neish AS, Read MA, Thanos D, Pine R, Maniatis T (1995). Endothelial IRF-1 cooperates with NF-κB as a transcriptional activator of vascular adhesion molecule-1.. Mol Cell Biol.

[14] Lewis H, Kaszubska W, DeLamarter JF, Whelan J (1994). Cooperativity between two NF-κB complexes, mediated by high-mobility-group protein I(Y) is essential for cytokine-induced expression of the E-selectin promoter.. Mol Cell Biol.

[15] Collins T, Read MA, Neish AS, Whitley MA, Thanos D (1995). Transcriptional regulation of endothelial cell adhesion molecules: NF-κB and cytokine-inducible enhancers.. FASEB J.

[16] Carvajal, IM, Baron RM, Perrella MA (2002). High mobility group-I/Y proteins: Potential role in the pathophysiology of critical illness.. Crit Care Med.

[17] Gotsch U, Jäger U, Dominis M, Vestweber D (1994). Expression of P-selectin on endothelial cells is upregulated by LPS and TNF-alpha in vivo.. Cell Adhes Commun.

[18] Sanders WE, Wilson RW, Ballantyne CM, Beaudet A (1992). Molecular cloning and analysis of in vivo expression of murine P-selectin.. Blood.

[19] Weller A, Isenmann S, Vestweber D (1992). Cloning of the mouse endothelial selectins. Expression of both E- and P-selectin is inducible by tumor necrosis factor alpha.. J Biol Chem.

[20] Thanos D, Maniatis T (1995). Virus induction of the human IFN-β gene expression requires the assembly of an enhanceosome.. Cell.

[21] Wolffe AP (1994). Architectural transcription factors.. Science.

[22] Reeves R, Nissen MS (1990). The A-T-DNA-binding domain of mammalian high mobility group I chromosomal proteins: A novel peptide motif for recognizing DNA structure.. J Biol Chem.

[23] Du W, Thanos D, Maniatis T (1993). Mechanisms of transcriptional synergism between distinct virus-inducible enhancer elements.. Cell.

[24] Thanos D, Maniatis T (1992). The high mobility group protein HMG I(Y) is required for NF-κB-dependent virus induction of the human IFN-β gene.. Cell.

[25] Perrella MA, Pellacani A, Wiesel P, Chin MT, Foster LC (1999). High mobility group-I(Y) protein facilitates nuclear factor-κB binding and transactivation of the inducible nitric-oxide synthase promoter/enhancer.. J Biol Chem.

[26] Pellacani A, Chin MT, Wiesel P, Ibanez M, Patel A (1999). Induction of high mobility group-I(Y) protein by endotoxin and interleukin-1β in vascular smooth muscle cells.. J Biol Chem.

[27] Cozzi P (2000). Recent outcome in the field of distamycin-derived minor groove binders.. Il Farmaco.

[28] Bell A, Kittler L, Lober G, Zimmer C (1997). DNA binding properties of minor groove binders and their influence on the topoisomerase II cleavage reaction.. J Mol Recognit.

[29] Bewley CA, Gronenborn AM, Clore GM (1998). Minor groove-binding architectural proteins: structure, function, and DNA recognition.. Annu Rev Biophys Biomol Struct.

[30] Huth JR, Bewley CA, Nissen MS, Evans JN, Reeves R (1997). The solution structure of an HMG-I(Y)-DNA complex defines a new architectural minor groove binding motif.. Nat Struct Biol.

[31] Wegner M, Grummt F (1990). Netropsin, distamycin, and berenil interact differentially with a high-affinity binding site for the high mobility group protein HMG-I.. Biochem Biophys Res Commun.

[32] Radic MZ, Saghbini M, Elton TS, Reeves R, Hamkalo BA (1992). Hoechst 33258, distamycin A, and high mobility group I (HMG-I) compete for binding to mouse satellite DNA.. Chromosoma.

[33] Baron RM, Carvajal IM, Liu X, Okabe RO, Fredenburgh LE (2004). Reduction of nitric oxide synthase-2 expression by distamycin A improves survival from endotoxemia.. J Immunol.

[34] Baron RM, Carvajal IM, Fredenburgh LE, Liu X, Porrata Y (2004). Nitric oxide synthase-2 down-regulates surfactant protein-B expression and enhances endotoxin-induced lung injury in mice.. FASEB J.

[35] Yoshizumi M, Hsieh CM, Zhou F, Tsai JC, Patterson C (1995). The ATF site mediates downregulation of the cyclin A gene during contact inhibition in vascular endothelial cells.. Mol Cell Biol.

[36] Wang X, Zhue Y, Kim HP, Song R, Zargenar R (2004). Hepatocyte growth factor protects against hypoxia/reoxygenation-induced apoptosis in endothelial cells.. J Biol Chem.

[37] Read MA, Whitley MZ, Gupta S, Pierce JW, Best J (1997). Tumor necrosis factor α-induced E-selectin expression is activated by the nuclear factor-κB and c-JUN N-terminal kinase/p38 mitogen-activated protein kinase pathways.. J Biol Chem.

[38] Himes SR, Reeves R, Attema J, Nissen M, Li Y (2000). The role of high-mobility group I(Y) proteins in expression of IL-2 and T cell proliferation.. J Immunol.

[39] Kozak M (1987). An analysis of 5′-noncoding sequences from 699 vertebrate messenger RNAs.. Nucl Acids Res.

[40] Takamiya R, Baron RM, Yet S-F, Layne MD, Perrella MA (2008). High mobility group A1 protein mediates human nitric oxide synthase 2 gene expression.. FEBS Lett.

[41] SenBanerjee S, Lin Z, Atkins GB, Greif DM, Rao RM (2004). KLF2 is a novel transcriptional regulator of endothelial proinflammatory activation.. J Exp Med.

[42] Pellacani A, Wiesel P, Razavi S, Vasilj V, Feinberg MW (2001). Down-regulation of high mobility group-I(Y) protein contributes to the inhibition of nitric-oxide synthase 2 by transforming growth factor-β1.. J Biol Chem.

[43] Grant MA*, Baron RM*, Macias AA, Layne MD, Perrella MA (2009). Netropsin improves survival from endotoxemia by disrupting HMGA1 binding to the NOS2 promoter.. Biochem J.

[44] Wang Y*, Baron RM*, Zhu G, Joo M, Christman JW (2006). PU.1 regulates cathepsin S expression in professional APCs.. J Immunol.

[45] Reeves R, Nissen MS (1999). Purification and assays for high mobility group HMG-I(Y) protein function.. Methods Enzymol.

[46] Weninger W, Carlsen HS, Goodarzi M, Moazed F, Crowley MA (2003). Naïve T cell recruitment to nonlymphoid tissues: A role for endothelium-expressed CC chemokine ligand 21 in autoimmune disease and lymphoid neogenesis.. J Immunol.

[47] Ejima K, Layne MD, Carvajal IM, Kritek PA, Baron RM (2003). Cyclooxygenase-2 deficient mice are resistant to endotoxin-induced inflammation and death.. FASEB J.

[48] Andonegui G, Bonder CS, Green F, Mullaly SC, Zbytnuik L (2003). Endothelium-derived Toll-like receptor-4 is the key molecule in LPS-induced neutrophil sequestration into lungs.. J Clin Invest.

[49] Coughlan AF, Hau H, Dunlop LC, Berndt MC, Hancock WW (1994). P-selectin and platelet-activating factor mediate initial endotoxin-induced neutropenia.. J Exp Med.

[50] Kamochi M, Kamochi F, Kim YB, Sawh S, Sanders JM (1999). P-selectin and ICAM-1 mediate endotoxin-induced neutrophil recruitment and injury to the lung and liver.. Am J Physiol (Lung Cell Mol Physiol).

[51] Klintman D, Li X, Thorlacius H (2004). Important role of P-selectin for leukocyte recruitment, hepatocellular injury, and apoptosis in endotoxemic mice.. Clin Diag Lab Immunol.

[52] von Andrian UH, Mackay CR (2000). T-cell function and migration. Two sides of the same coin.. N Engl J Med.

[53] Bevilacqua MP, Pober JS, Mendrick DL, Cotran RS, Gimbrone MA (1987). Identification of an inducible endothelial-leukocyte adhesion molecule.. Proc Natl Acad Sci USA.

[54] Mayadas TN, Johnson RC, Rayburn H, Hynes RO, Wagner DD (1993). Leukocyte rolling and extravasation are severely compromised in P Selectin-deficient mice.. Cell.

[55] Ghersa P, Whelan J, Cambet Y, DeLamarter JF, van Huijsduijnen RH (1997). Distamycin prolongs E-selectin expression by interacting with a specific NF-[kappa]B-HMG-I(Y) binding site in the promoter.. Nucl Acids Res.

[56] Steeber, DA, Tang MLK, Green NE, Zhang X-Q, Sloane JE (1999). Leukocyte entry into sites of inflammation requires overlapping interactions between the L-selectin and ICAM-1 pathways.. J Immunol.

[57] Frenette PS, Mayadas TN, Rayburn H, Hynes RO, Wagner DD (1996). Susceptibility to infection and altered hematopoiesis in mice deficient in both P- and E-selectins.. Cell.

[58] Basit A, Reutershan J, Morris MA, Solga M, Rose CE (2006). ICAM-1 and LFA-1 play critical roles in LPS-induced neutrophil recruitment into the alveolar space.. Am J Physiol Lung Cell Mol Physiol.

[59] Xu H, Gonzalo JA, Pierre Y St, Williams IR, Kupper TS (1994). Leukocytosis and resistance to septic shock in intercellular adhesion molecule-1 deficient mice.. J Exp Med.

[60] Kamochi M, Kamochi F, Kim YB, Sawh S, Sanders JM (1999). P-selectin and ICAM-1 mediate endotoxin-induced neutrophil recruitment and injury to the lung and liver.. Am J Physiol Lung Cell Mol Physiol.

[61] Steeber DA, Campbell MA, Basit A, Ley K, Tedder TF (1998). Optimal selectin-mediated rolling of leukocytes during inflammation in vivo requires intercellular adhesion molecule-1 expression.. Proc Natl Acad Sci.

[62] Geng J-G, Cheng M, Chou K-C (2004). P-selectin cell adhesion molecule in inflammation, thrombosis, cancer, growth, and metastasis.. Current Med Chem.

[63] Hickey MJ, Granger DN, Kubes P (2001). Inducible nitric oxide synthase (iNOS) and regulation of leucocyte/endothelial cell interactions: studies in iNOS-deficient mice.. Acta Physiol Scand.

[64] Zarbock A, Singbartl K, Ley K (2006). Complete reversal of acid-induced acute lung injury by blocking of platelet-neutrophil aggregation.. J Clin Invest.

[65] Asaduzzaman M, Rahman M, Jeppsson B, Thorlacius H (2009). P-selectin glycoprotein-ligand-1 regulates pulmonary recruitment of neutrophils in a platelet-independent manner in abdominal sepsis.. British J Pharm.

[66] Janssen S, Durussel T, Laemmli UK (2000). Chromatin opening of DNA satellite by targeted sequence-specific drugs.. Mol Cell.

[67] Janssen S, Cuvier O, Müller M, Laemmli UK (2000). Specific gain- and loss-of-function phenotypes induced by satellite-specific DNA-binding drugs fed to *Drosophila melanogaster*.. Mol Cell.

[68] Henikoff S, Vermaak D (2000). Bugs on drugs go GAGAA.. Cell.

[69] Susbielle G, Blattes R, Brevet V, Monod C, Käs E (2005). Target practice: Aiming at satellite repeats with DNA minor groove binders.. Curr Med Chem—Anti-Cancer Agents.

[70] Baraldi PG, Preti D, Fruttarolo F, Tabrizi MA, Romagnoli R (2007). Hybrid molecules between distamycin A and active moieties of antitumor agents.. Bioorg and Med Chem.

[71] Doss RM, Marques MA, Foister S, Chenoweth DM, Dervan PB (2006). Programmable oligomers for minor groove DNA recognition.. J Am Chem Soc.

[72] Dolenc J, Baron R, Oostenbrink C, Koller J, van Gunsteren WF (2006). Configurational entropy change of netropsin and distamycin upon DNA minor-groove binding.. Biophys J.

[73] Dolenc J, Oostenbrink C, Koller J, van Gunsteren WF (2005). Molecular dynamics simulations and free energy calculations of netropsin and distamycin binding to an AAAAA DNA binding site.. Nucl Acids Res.

[74] Baraldi PG, Bovero A, Fruttarolo F, Preti D, Tabrizi MA (2004). DNA minor groove binders as potential antitumor and antimicrobial agents.. Med Res Rev.

[75] Thomas R, Gonzalez C, Roberts C, Botyanszki J, Lou L (2004). A novel assay to determine the sequence preference and affinity of DNA minor groove binding compounds.. Nucl Acids Res.

[76] Xie X, Lu J, Kulbokas EJ, Golub TR, Mootha V (2005). Systematic discovery of regulatory motifs in human promoters and 3′ UTRs by comparison of several mammals.. Nature.

